# Supplementary figures and images for: TML1 and TML2 synergistically regulate nodulation and affect arbuscular mycorrhiza in Medicago truncatula
Source: Front Plant Sci. 2024 Dec 11;15:1504404. doi: 10.3389/fpls.2024.1504404 (PMC11668588; doi:10.3389/fpls.2024.1504404)

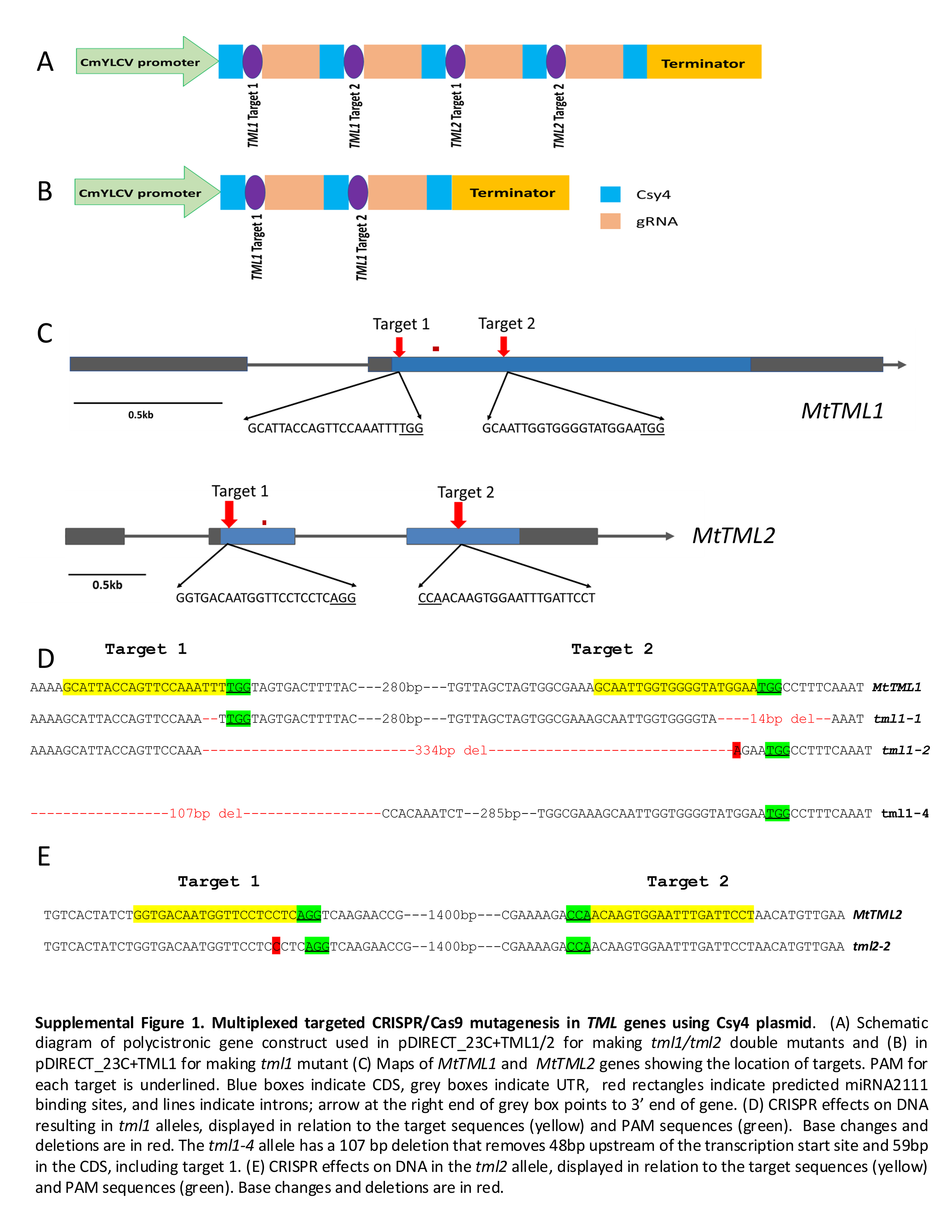

Supplement: Supplementary Figure 1 — Multiplexed targeted CRISPR/Cas9 mutagenesis in TML genes using Csy4 plasmid. (A) Schematic diagram of polycistronic gene construct used in pDIRECT_23C+TML1/2 for making tml1/tml2 double mutants and (B) in pDIRECT_23C+TML1 for making tml1 mutant (C) Maps of MtTML1 and MtTML2 genes showing the location of targets. PAM for each target is underlined. Blue boxes indicate CDS, grey boxes indicate UTR, red rectangles indicate predicted miRNA2111 binding sites, and lines indicate introns; arrow at the right end of grey box points to 3’ end of gene. (D) CRISPR effects on DNA resulting in tml1 alleles, displayed in relation to the target sequences (yellow) and PAM sequences (green). Base changes and deletions are in red. The tml1-4 allele has a 107 bp deletion that removes 48bp upstream of the transcription start site and 59bp in the CDS, including target 1. (E) CRISPR effects on DNA in the tml2 allele, displayed in relation to the target sequences (yellow) and PAM sequences (green). Base changes and deletions are in red. [file Image1.tif]
